# Five Quantitative Trait Loci Control Radiation-Induced Adenoma Multiplicity in *Mom1^R^ Apc^Min/+^* Mice

**DOI:** 10.1371/journal.pone.0004388

**Published:** 2009-02-05

**Authors:** Eiram Elahi, Nirosha Suraweera, Emmanouil Volikos, Jackie Haines, Natalie Brown, Gerovie Davidson, Mike Churchman, Mohammed Ilyas, Ian Tomlinson, Andrew Silver

**Affiliations:** 1 Colorectal Cancer Genetics, Institute for Cell and Molecular Sciences, Barts and The London, Queen Mary's School of Medicine and Dentistry, London, United Kingdom; 2 HPA Radiation Protection Division, Chilton, Didcot, Oxon, United Kingdom; 3 Cancer Research UK Genotyping Facility, Clinical Pharmacology, University of Oxford, Radcliffe Infirmary, Oxford, United Kingdom; 4 School of Molecular Medical Sciences, Division of Pathology, Queen's Medical Centre, Nottingham, United Kingdom; 5 Molecular and Population Genetics Laboratory, London Research Institute, Cancer Research UK, London, United Kingdom; National Institute on Aging, United States of America

## Abstract

Ionising radiation is a carcinogen capable of inducing tumours, including colorectal cancer, in both humans and animals. By backcrossing a recombinant line of *Apc^Min/+^* mice to the inbred BALB/c mouse strain, which is unusually sensitive to radiation–induced tumour development, we obtained panels of 2Gy-irradiated and sham-irradiated N2 *Apc^Min/+^* mice for genotyping with a genome-wide panel of microsatellites at ∼15 cM density and phenotyping by counting adenomas in the small intestine. Interval and composite interval mapping along with permutation testing identified five significant susceptibility quantitative trait loci (QTLs) responsible for radiation induced tumour multiplicity in the small intestine. These were defined as *Mom* (Modifier of *Min*) radiation-induced polyposis (*Mrip*1-5) on chromosome 2 (log of odds, LOD 2.8, p = 0.0003), two regions within chromosome 5 (LOD 5.2, p<0.00001, 6.2, p<0.00001) and two regions within chromosome 16 respectively (LOD 4.1, p  = 4×10^−5^, 4.8, p<0.00001). Suggestive QTLs were found for sham-irradiated mice on chromosomes 3, 6 and 13 (LOD 1.7, 1.5 and 2.0 respectively; p<0.005). Genes containing BALB/c specific non-synonymous polymorphisms were identified within *Mrip* regions and prediction programming used to locate potentially functional polymorphisms. Our study locates the QTL regions responsible for increased radiation-induced intestinal tumorigenesis in *Apc^Min/+^* mice and identifies candidate genes with predicted functional polymorphisms that are involved in spindle checkpoint and chromosomal stability (*Bub1b, Casc5*, and *Bub1*), DNA repair (*Recc1* and *Prkdc*) or inflammation (*Duox2, Itgb2l* and *Cxcl5).* Our study demonstrates use of *in silico* analysis in candidate gene identification as a way of reducing large-scale backcross breeding programmes.

## Introduction

The genetic dissection of complex traits is a difficult and important challenge. A number of resistant and susceptibility quantitative trait loci (QTLs) for tumours arising in many tissues (lung, colon, skin and haemopoietic system) have been mapped using mice (1, 2). Unfortunately, relatively few QTLs have been translated into critical polymorphisms within genes that can be demonstrated to show a clear alteration in the risk of tumour development. The lack of a systematic approach to the selection of genes within QTLs coupled with extensive breeding programmes to define relatively small intervals without taking into account clusters of interacting genes has contributed to this disappointing return. The search of genes within QTLs has recently benefited from the advances in the mammalian genome research, particularly with regard to mouse strain-specific sequence databases allowing *in silico* discovery of gene-coding and gene-regulatory variants (3). This approach combined with bioinformatics, including software to interrogate polymorphisms for functional effects provide an effective method for discovering the genes behind multigenic diseases such as cancer.

By far the majority of tumours used for QTL mapping purposes have been induced using high doses of chemical carcinogens and mutagens (2). This raises the possibility that a number of the QTLs will reflect genes involved in xenobiotic metabolism and clearance from the body rather than *bona fide* modifiers of carcinogenesis. Furthermore, the majority of the general human population are unlikely to be exposed to the chemicals used in these studies outside the context of occupational exposure. Consequently, it remains to be seen whether any QTLs identified through chemical tumorigenesis studies have homologues relevant to the appearance of cancer in humans. A complementary approach is to use rodent models where the primary genetic event leading to tumorigenesis is already known, and then to map modifiers of disease severity through selective breeding. The best example of this rationale involves the use of the multiple intestinal neoplasia (*Min*) mice, the first mouse model system involving the adenomatous polyposis coli (*Apc*) gene. *Apc^Min/+^* mice develop numerous intestinal adenomas and are a good model of human familial adenomatous polyposis (FAP). The model has provided examples of modifying loci (called modifiers of *Min*, *Mom*) in mice, demonstrating the principle of genetic modulation of disease severity represented by, in these cases, intestinal adenoma multiplicity. To date, the chromosomal positions of *Mom1-3*, *Mom6* and *Mom7* have been reported (4) and functional polymorphisms identified for *Mom1* and *2* (5).

Ionizing radiation is a well-characterized carcinogen capable of inducing tumours, including colorectal cancer (CRC), in both humans and animals (6). All humans are exposed to radiation as a consequence of environmental background; occupational and medical radiation sources provide additional hazards. For example, some 6,000 patients undergo pelvic radiotherapy each year and many show inflammatory responses in the gut, a known risk factor for CRC (7). Using a recombinant line of *Apc^Min/+^* mice and the BALB/c mouse strain, which is unusually sensitive to ionising radiation–induced tissue damage and tumour development, we have previously shown that adenoma multiplicity in irradiated *Apc^Min/+^* mice can be modified by two QTLs, which map to chromosome 16 segments from BALB/c (8). We have now undertaken the first genome-wide scan for additional loci modifying radiation-induced tumour multiplicity in the intestine of N2 backcross *Min* mice. The successful identification of candidate genes for QTLs using a combination of *in silico* and bioinformatics demonstrates the effectiveness of our approach.

## Results

### Identification of main effect QTLs controlling adenoma multiplicity

We have conducted a genome-wide study involving microsatellite genotyping of constitutional DNA from panels of 2 Gy x-irradiated (84 males and 58 females) and sham-irradiated (60 males and 51 females) N2 *Apc^Min/+^* mice. Each N2 mouse was genotyped using a total of 112 microsatellites with an average interval space between markers of 15 cM based on Kosambi's distances created by the MapManager QTX programme. Following our earlier observations on *Apc^Min/+^* mice that the upper part of the small intestine (duodenum and jejunum) was more sensitive than the lower (ileum) region to radiation-induced adenoma formation (8) and that there may be modifiers that influence tumour multiplicity in the different parts, animals were assessed for adenoma multiplicity in the upper (USI) and lower small intestine (LSI) at time of sacrifice (all mice >80 days; Mean: 0 Gy, 241 days; 2 Gy, 180 days). The data for USI and LSI were analysed separately. Interval mapping (IM) and permutation testing (PT) were used to determine threshold values for each trait and an overall threshold value of 12.6 likelihood ratio statistic (LRS) was calculated from over 18 separate PTs (P<0.05); empirical p values were calculated for the observed LRS values (Table 1).

**Table 1:** Main effect QTLs for tumour multiplicity in the small intestine of mice identified using a panel of 142 N2 irradiated (2Gy) mice.

Chr. (region)	Trait	Locus (cM)	p-value (LRS)	%	±CI	Association
Chr.2 (*Mrip1*)	USI	*D2Mit395* (55.7)	3.2×10^−4^ (12.9)	9	21.5	Si (≥12)
Chr.5 (*Mrip2*)	USI	*D5Mit201* (28.4)	<0.00001 (24)	15	13.5	HSi (≥20.2)
Chr.5 (*Mrip3*)	USI	*D5Mit139* (59.0)	<0.00001 (28.6)	19	10	HSi (≥20.2)
Chr.16 (*Mrip4*)	USI	*Prkdc* (9.2)	4×10^−5^ (19)	11	16.5	Si (≥12.6)
Chr.16 (*Mrip5*)	USI	*D16Mit189* (40.1)	<0.00001 (22.2)	14	13.5	Si (≥12.6)
Chr.17	USI	*D17Mit180* (25.1)	8.2×10^−3^ (7)	5	39	Su (≥6.76)
Chr.1	LSI	*D1Mit17* (110.4)	4.4×10^−3^ (8.6)	6	33.5	Su (≤12)
Chr.6	LSI	*D6Mit194* (51.4)	2.6×10^−3^ (8.8)	6	30	Su (≤12.8)
Chr.7	LSI	*D7Mit178* (0.0)	7×10^−2^ (12.2)	6	38	Su (≤12.5)
Chr.8	LSI	*D8Mit211* (5.3)	0.007 (9.5)	5	38	Su (≤12.7)
Chr.11	LSI	*D11Mit216* (14.2)	6.5×10^−3^ (7.6)	7	37	Su (≤12.1)

The chromosome (Chr.) and region defined as *Mrip (Mom* radiation-induced polyposis) are shown in column 1, with the traits; USI (upper small intestine) and LSI (lower small intestine) in column 2. The marker name and position are determined by MIT; (http://www.broad.mit.edu/cgi-bin/mouse/sts_info?databasemouserelease) and shown in column 3. Also given are the p-values with LRS (Likelihood Ratio Statistic), the percentage trait variance (%) and confidence intervals (CI). The level of significance for each association are categorised as suggestive (Su), significant (Si) or highly significant (HSi) based on LRS (value ≥ given in parenthesis).

**Figure 1:**
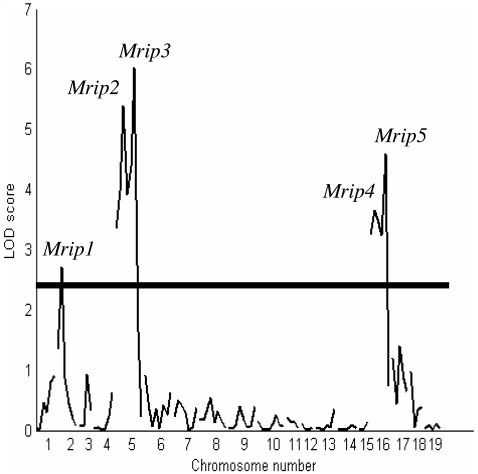
A genome wide scan to show the LOD profiles of chromosomes 1–19 with adenoma multiplicity in the USI of the 2 Gy cohort. The horizontal line above the *y* axis represents a significant threshold score. Significant QTLs are detected on chromosomes 2, 5 and 16 and are defined as *Mom* radiation-induced polyposis (*Mrip*1–5; arrowed).

In addition to the previously reported QTLs on chromosome 16 (p<0.0001; 8), we found two highly significant QTLs (p<0.0001; Table 1; Fig. 1) on chromosome 5, one significant QTL on chromosome 2, (p<0.0001; Table 1; Fig. 1) and one suggestive QTL on chromosome 17 for USI trait (p<0.001; Table 1). The two QTLs on chromosome 5 explained 15% and 19% of the total trait variance, the two QTLs on chromosome 16 accounted for 11% and 14% and chromosome 2 explained 9%. Overall, these significant QTLs of the USI account for 68% trait variation and requires further investigation. Only suggestive QTLs were identified for the LSI on chromosomes 1, 6, 7, 8 and 11 (p<0.01; Table 1). The 0 Gy dataset yielded only suggestive QTLs on chromosomes 3 and 13 for the USI trait and on chromosome 6 for the LSI (Table 2). We define the most significant QTLs (Table 1 and Fig. 1) as *Mom* radiation-induced polyposis (*Mrip*) loci. To confirm the existence of two independent USI QTLs on chromosome 5, mice were genotyped using an additional marker *D5Mit139* (69.0 cM) and the IM and PT re-run. This revealed a substantial QTL effect (LRS of 28.8; P<0.0001) at the beginning of the interval defined by *D5mit139* and the next proximal marker, *D5Mit188* (64 cM). This locus retained its strong association (highly significant at p<0.0001) following further PT using 10,000 scans (chromosomes 1–19). To confirm results from MapManager, we repeated IM analysis for the irradiated cohort of mice using a compatible format Cartographer QTL Version 1.17 (9,10). PT (1,000 tests; 2cM window) with all markers for each of the four traits for 2Gy dataset provided similar LRS threshold values as MapManager. The only exception was *D2Mit395* (LOD 2.7), which showed borderline significance when analysed using MapManager, but with Cartographer the marker fell just below the significance threshold of LOD 2.8.

**Table 2:** Main effect QTLs for tumour multiplicity in the small intestine of mice identified using a panel of 112 N2 sham-irradiated mice.

Chr.	Trait	Locus (cM)	p-value (LRS)	%	±CI	Association
Chr. 3	USI	*D3Mit352* (63.4)	3.5×10^−4^ (8)	9	±32.5	Su (≤11.6)
Chr. 13	USI	*D13Mit179* (15.3)	3.7×10^−4^ (9.2)	8	±33	Su (≤11.8)
Chr. 6	LSI	*D6Mit83* (3.3)	6.5×10^−4^ (7.1)	7	±39	Su (≤12.3)

The first column shows the chromosome (Chr.) number with the traits; USI (upper small intestine) and LSI (lower small intestine) in column 2. The marker name and position (cM) are determined by MIT; (http://www.broad.mit.edu/cgi-bin/mouse/sts_info?databasemouserelease) and shown in column 3. Also given are the p-values with LRS (Likelihood Ratio Statistic), the percentage trait variance (%) and confidence intervals (CI). The level of significance attained was suggestive (Su) for all QTLS of the 0 Gy cohort.

**Figure 2 pone-0004388-g002:**
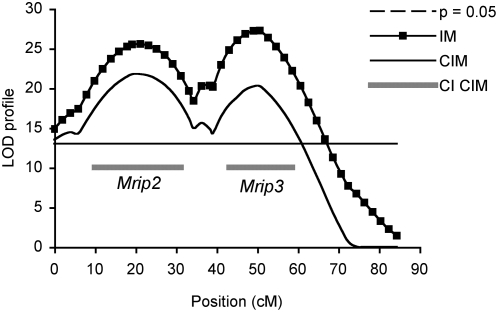
The LRS profile of *Mrip2* and *Mrip3* against markers distributed along chromosome 5 is determined using CIM (composite interval mapping) and compared with IM (interval mapping). The horizontal bar represents genome wide significance at p = 0.05. *Mrip2* and *Mrip3* are confirmed as separate non-linked QTLs associated with adenoma multiplicity in the USI of the 2 Gy mice.

**Figure 3:**
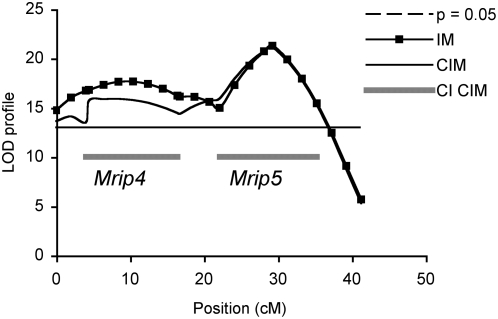
The LRS profile of *Mrip4* and *Mrip5* against markers distributed along chromosome 16 is determined using CIM (composite interval mapping) and compared with IM (interval mapping). The horizontal bar represents genome wide significance at p = 0.05. *Mrip4* and *Mrip5* are confirmed as separate non-linked QTLs associated with adenoma multiplicity in the USI of the 2 Gy mice.

Composite Interval Mapping (CIM; (11,12)) was used to assess any background or ‘ghost’ effects associated with the multiple QTLs found on both chromosome 5 and 16. A test window of 30 cM was selected based on genetic distance between the two QTLs on chromosome 5 (*D5Mit201* and *D5Mit139*; 30 cM) and on 16 (*Prkdc* and *D16Mit189*; 20 cM). A maximum of five significant markers outside of the test window, were selected as background controls and fitted to a forward and reverse regression models as coefficients to control for genetic variation (13). The number of markers selected was sufficient to account for background QTL effects, and both IM and CIM were equally efficient in detecting QTLs with large effects on chromosomes 5 and 16. When compared with CIM, IM tended to have the highest power of detecting QTLs at *D5Mit201*, *D5Mit139*, and at *Prkdc*; there was no difference between mapping methods for *D16Mit189.* Importantly, no false QTLs were detected under the threshold values, and both IM and CIM gave similar likelihood findings. These analyses confirmed that the two peaks observed on chromosomes 5 and on 16 are independent QTLs with strong effects (Fig. 2 and 3 respectively). We were now able to confirm *Mrips* as five independent loci (*Mrip*1–5; Table 1; Fig. 1–3) influencing adenoma multiplicity in the USI of *Min* recombinant mice.

### Selection of genes within QTL regions

We focussed on the five most significant main effect QTLs, *Mrip*1–5. Gene lists were drawn up for the chromosomal segment up and down stream (±15 cM) of the most likely position for each QTL (Table 3). The markers for each peak were: for chromosome 2, *D2Mit395* (*Mrip1*); for chromosome 5, *D5Mit139* (*Mrip2*) and *D5Mit201* (*Mrip3*); for chromosome 16, *D16Mit189* (*Mrip4*) and *Prkdc* (*Mrip5*). Interestingly, *Mrip5* contained the *Ritls* loci associated with radiation induced thymic lymphoma (14) and the *Mrip1* region encompasses the *Scc* (Colon tumour susceptibility) *1* and *2* loci associated with colon cancer susceptibility (15). For all QTLs combined, a total of 373 genes containing coding non-synonymous single nucleotide polymorphism (CnSNPs; n = 678) were identified between the two inbred strains using the MPD database. Olfactory genes (n = 166) containing 138 CnSNPs were found within the *Mrips*, but were excluded from further analysis because of an assumption of lack of functional relevance. Genes from this list was further selected based on their involvement in tumorigenesis, inflammatory response and radiation effects (Table 3). By using these processes we selected a number of genes for sequence verification in the BALB and *B6^Min/+^* line. In total, 14 genes from *Mrip1*, 14 genes from both *Mrip2* and *Mrip3*, and 11 genes from *Mrip4* and *Mrip5* were chosen for verification by sequencing (Table 3).

**Table 3:** Summary of the process used in selecting genes from QTL regions.

*Mrip* (Chromosome and QTL position)	Region (±15 cM QTL)	*In silico* Approach	CnSNPs	*In silico* Approach	Genes selected for further analysis	*In silico* Approach
*Mrip1* Chr. 2 55.7 cM	40 cM (69.2 Mbp) – 70 cM (131.6 Mbp)	Gene lists based on BALB specific SNPs using MPD	170	Genes are selected on the basis of their functional involvement in tumorigenesis, inflammation and radiation response	*Rad51, AK1* *Il1-beta, Ilrn* *Duox2, Bubr1* *Thbs1, Mertk* *Dll4, Bub1* *Casc5, ITGA6* *Madd, Cat*	**Molecular analysis of SNPs** Sequence confirmation **Functional prediction of SNPs**
*Mrip2* Chr. 5 28.4 cM	13.4 cM (21.3 Mbp) – 43.4 cM (85.1 Mbp)		43		*Pdgfra* *Il-6* *Kit* *Xrcc2* *Centd1* *Recc1*	-amino acid conservation (EMBL-EBI) -Structure and function of SNPs (PolyPhen and SIFT)
*Mrip3* Chr. 5 59 cM	44 cM (90.4 Mbp) – 74cM (138.3 Mbp)		105		*Nos1* *Hmgb* *Rac1* *Tcf1* *Mmp17* *Usp42* *Cxcl5* *Cxcl9*	-functional domains (literature and STRING)
*Mrip4* Chr. 16 9.2 cM	0 cM (3.8 Mbp) – 25 cM (37.3 Mbp)		23		*Muc20* *Prkdc* *Muc4* *Mapk1* *Stch*	
*Mrip5* Chr. 16 40.1 cM	25 cM (37.3 Mbp) – 55 cM(84.7 Mbp)		32		*App* *Ets2* *Itgb2l* *Sod1* *Tiam1* *Hmgn1* *Runx1* *Stch*	

### Predicting functional mutations in selected genes using *in silico* analysis

#### Mrip1

A combination of bioinformatics programmes was used to identify possible functional mutations for the CnSNPs (Table 3). We identified possibly damaging mutations in six genes within the *Mrip1* region: *Duox2, Bubr1, Mertk, Dll4, Casc5 and Bub1* (Table 4). Three BALB specific single nucleotide polymorphisms (SNPs) were found in the *Duox2* gene: H627R has only partial conservation among species (36%), but was predicted to be a damaging mutation according to Polyphen with a position specific independent count (PSIC) of 1.524. The polymorphisms, S553G and N378S were located within the extra cellular, N terminal heam peroxidase domain, although both were not predicted to be damaging substitutions.

**Table 4:** Predictive assessment of *BALB* specific amino acid polymorphisms for potentially damaging alterations.

	Gene	Variation polymorphism	Functional domain	Polyphen (PSIC score)	SIFT	Conservation (%)
*Mrip1*	*Duox2*	H627R rs27453362		**1.524**	T	36% (9/25)
		G553S rs27453360	Animal heam peroxidase (a.a 60–562)	0.114	T	44% (11/25)
		N378S rs27453345		0.299	T	36% (9/25)
	*Bub1b*	S240G rs3144766	CDC20 binding to BUBR1 (Mad2 dependent)	1.32	T	75% (6/8)
		P278T rs3149979		0.549	T	44% (4/9)
		V312A rs3149980		0.072	T	63% (5/8)
		D664E rs27423851	CDC20 binding to BUBR1 (Mad2 independent)	1.241	N (Acidic to acidic)	67% (5/8)
		N705D Rs27423850		0.345	N (Acidic to uncharged polar)	67% (5/8)
	*Dll4*	Q419L rs27423750	EGF like 6	0.431	T	25% (15/59)
		G627S rs27423747		**1.516**	N (Uncharged polar to non-polar)	38% (6/16)
	*Casc5*	T1286I rs27471474		**1.643**	T	60% (3/5)
	*Bub1*	P153S Rs28269039		**1.569**	T	57% (4/7)
*Mrip2*	*Centd1*	R278P rs31438432		**2.271**	N (Basic to non-polar)	100% (7/7)
	*Recc1*	N66Y rs31457275		**2.440**	T	79% (8/11)
*Mrip3*	*Cxcl5*	R10H rs31723268		**1.582**	N/A	100% (2/2)
*Mrip4*	*Prkdc*	M3844V rs48580935	Nuc194 domain	0.208	T	50% (4/8)
		R2140C rs4164952		**2.495**	N (basic to uncharged polar)	100% (9/9
*Mrip5*	*Itgb2l*	D509N rs3166158		**2.193**	T	100% (45/45)
		G539S rs3164214		**1.662**	T	56% (25/45)

We list the polymorphisms using MPD (Mouse Phenome Database) and known functional domains using STRING (Search Tool for the Retrieval of Interacting Genes/Proteins) within the *Mrip* regions. Polyphen predictions are based on position specific independent count (PSIC) and if >1.5 then potentially damaging and shown in bold. SIFT predicts the substitution to be tolerated (T) or not tolerated (N), if intolerant then the substitution effect is stated. The conservation of sequence alignment at and around the amino acid substitution is compared using EMBL-EBI.

Two CnSNPs within *Dll4* were observed, including a substitution at G627S predicted to be damaging according to Polyphen analysis due to a significant hydrophobic substitution effect (PSIC score = 1.516). This substitution causes an uncharged polar molecule (S) to change into a non-polar molecule (G) at position 627 according to SIFT. This amino acid was not evolutionarily conserved (6/16) and did not belong to any known functional domain. Although the Q419L alteration was within the 3ed EGF-like domain, the mutation itself was predicted to not cause any functional alterations to the protein's activity (Table 4).

Five CnSNPs were detected within *Bub1b*, three of which (S240G, P278T and V312A) are within a Mad2 dependent, Cdc20 binding domain necessary for effective cell cycle checkpoint functions (16). With regard to the other two CnSNPs, both SIFT and Polyphen analysis indicated that all these substitutions, although within functional domains, were tolerated. *Casc5* and *Bub1* polymorphisms (T1285I and P152S, respectively) were predicted to be deleterious (PSIC>1.5), although none were within any known functional domains.

#### *Mrip2* and *Mrip3*

Possible functional CnSNPs were identified for *Centd1*, *Recc1* and *Cxcl5*. The *Centd1,* and *Cxcl5* polymorphisms (R278 and R10H, respectively) were found in regions of the proteins that were highly conserved between different vertebrates whilst the N66Y polymorphism from *Recc1* was only partially conserved at 79%. Although these regions in which these polymorphisms were found did not form part of any known functional domains, the R278P, R10H and Y66N were not tolerated substitutions according to Polyphen predictions (PISC = 1.582). The change from a basic molecule, R, to the non-polar molecule, P, in the R278P *Centd1* polymorphism was predicted ‘probably damaging’ (PSIC = 2.270) and ‘intolerant’ using Polyphen and SIFT respectively. The N66Y polymorphism of *Recc1* was also predicted ‘probably damaging’ (PSIC = 2.44) using Polyphen, but ‘tolerant’ according to SIFT. All the protein prediction analyses indicated that these substitutions could be functionally important.

#### *Mrip4* and *Mrip5*

*Prkdc* is the most likely candidate for *Mrip4* (8,14,17,18,19). Of the two identified *Prkdc* polymorphisms, R2140C involves the substitution of a basic molecule (R) to an uncharged polar molecule (C) which was predicted as damaging (PSIC = 2.495) using Polyphen and intolerant according to SIFT analysis. The amino acid sequence is highly conserved with invertebrates and forms part of the Nuc194 domain involved in DNA repair (Table 4). Within the *Mrip5* region the *Itgb2l* gene on chromosome 16 has two substitutions, D509N and G539S, and both polymorphisms were considered to be deleterious according to Polyphen (PSIC = 2.193 and 1.628, respectively). We also found that the D509 amino acid residue was conserved in all vertebrates (100%; 11/11) for which sequence data was available (Table 4).

## Discussion

In this study of a recombinant *Apc^Min/+^ BALB/cByJ* mouse model we have identified five significant QTLs associated with intestinal tumour multiplicity in the USI. The sham irradiated cohort of mice yielded only suggestive QTLs for the USI and the LSI trait. Of the three suggestive spontaneous QTLs none map to chromosome 18 suggesting that *Mom2*, 3 and 7 are not involved in determining adenoma multiplicity in this genetic background or only have very weak influences that would require substantially more mice to investigate. Also, these loci may not have a role in this study because of the genetic background used. For instance, *Mom2* is a spontaneous mutation not carried by the mice used here and the recessive allele conferring susceptibility to severe disease (*Mom3*) is not present. There are a number of alleles of *Mom7* segregating amongst mouse strains, but unfortunately BALB has not been investigated to date (4); our data would suggest that this loci has no, or a very weak, influence on adenoma multiplicity in the mice used here. Of the main effects radiation associated QTLs investigated in this paper, *Mrip2* and *Mrip3* showed the most significant association with tumour multiplicity followed by *Mrip1*, *Mrip4* and *Mrip5*. *In silico* and predictive methodologies were used to identify possible genes underlying these QTLs. With advances in whole genome databases, and the availability of near complete sequence data on different strains of mice, it is now possible to use *in silico* data analysis to identify the genes that map within delineated chromosomal intervals and to find any variations in amino acids sequence in candidate genes (20). Hence, QTL mapping in conjunction with *in silico tools,* including subsequent protein predictive programming to assign a functional role, can provide a powerful and relatively robust means of identifying susceptibility genes in multigene diseases such as tumorigenesis (Tables 4 and 5).

**Table 5:** The function of candidate genes and their role in radiation response and tumorigenesis.

Symbol	Gene	Functional/expression
*Duox2*	Dual oxidase 2	Generate ROS species; Innate immune response; expressed in mucosal surfaces including gut (30); hypermethylated in lung cancer (31).
*Bub1b*	Budding uninhibited by benzimidazoles 1 homolog, beta	Mitotic checkpoint gene involved in chromosomal instability. Upregulation in CRC (32).
*DLL4*	Delta-like 1	Notch signalling, regulates tumour angiogenesis and tumour growth, expressed in the gut (33).
*Casc5*	Cancer susceptibility candidate 5, AF15q14	A member of the *Spc105/Spc7/KNL-1* family, directly links spindle checkpoint proteins *Bub1b* and *Bub1* to kinetochores and is required for chromosome alignment (34)
*Bub1*	Budding uninhibited by benzimidazoles 1 homolog	Regulates exit from cell cycle checkpoint, genetic and epigenetic alterations in CRC (35)
*Centd1*	Centaurin, delta 1	Human homologue involved in focal adhesion and mediates effects of *RhoA* (36)
*Recc1*	Replication factor C (activator 1) 1	A multi-subunit protein complex needed for proliferating cell nuclear antigen (*PCNA*)-dependent DNA replication and repair synthesis (37).
*Cxcl5*	Chemokine (C-X-C motif) ligand 5	Chemokines as regulators of angiogenesis (38)
		Expressed in the colon and variants associated with CRC (39).
*Prkdc*	Protein kinase, DNA activated, Catalytic polypeptide	DNA double strand break repair mechanism (40).
*Itgb2l*	Integrin beta 2-like	During an inflammatory response, *Itg2l* help retain *Cxcl13*-expressing cells (41)

Our approach has identified ten candidate genes, most of which are involved in spindle checkpoint and chromosomal stability (*Bub1b, Casc5*, and *Bub1*), DNA repair (*Recc1* and *Prkdc*) or inflammation (*Duox2, Itgb2l* and *Cxcl5*; Table 5). *Dll4* is a key player in the Notch signalling pathway known to regulate tumour angiogenesis, as does *Cxcl5*, and growth (Table 5). The role of *Centd1* is not understood, although the protein functions down stream of RhoA to regulate focal adhesion dynamics suggesting an involvement in invasion and metastasis in cancer (21). Our results might suggest other roles for this protein in tumorigenesis and further functional tests.

From our study it is evident that QTLs can be systematically searched to identify possible candidate genes responsible for disease by using a combination of *in silico* methods, although it should be cautioned that gene selection at any given time would be based on genes already known or suspected to be involved in tumorigenesis. In addition to CnSNPs and SSNPs studied in this paper there are many other BALB specific SNPs within these QTL regions. Some of these SNPs are situated within regulatory domains outside the gene coding regions such as promoters, enhances and untranslated regions (UTR)s. These SNPs may ultimately influence the regulation of gene expression. ExpressionQTL (eQTL) analysis, an alternative *in silico* approach, provides a means of identifying regulatory SNPs associated with the expression of genes located within QTLs (22). We will employ eQTL analysis in the future to identify *BALB* specific regulatory SNPs associated with radiation induced tumorigenesis in the *Apc^Min/+^ BALB/cByJ* mouse model.

In summary, we have identified five novel QTLs controlling susceptibility to radiation-induced polyposis in *Apc^Min/+^* mice. By using an effective *in silico* approach, we were able to identify potential candidate genes within these QTLs. The functional influence of these polymorphisms within our candidate genes will require substantial *in vivo* and *in vitro* investigations.

## Materials and Methods

### Animals, adenoma induction and Scoring

A recombinant inbred line (Line I) of *Apc^Min/+^* mice on a C57BL/6 background (B6*^Min/+^*) that showed limited intraline variation in adenoma numbers was established and maintained as detailed previously (23). Female BALB/cByJ (BALB) mice were crossed with male B6*^Min/+^* to yield F1 (N1) progeny. N1 male *Apc^Min/+^* were then backcrossed to female BALB to produce N2 offspring that carry at least one copy of the BALB-derived modifier of *Min* (*Mom1*) dominant resistance allele (*Mom1^R^*). Irradiations and sham-irradiations were performed as given previously on N2 *Apc^Min/+^* mice (8). Animals were housed in conventional cages with water, and standard maintenance diet, provided *ad libitum*. Mice were killed by CO_2_ asphyxiation when quality of life was compromised. Intestinal tracts were prepared for adenoma counting as detailed earlier (8,23). The small intestine was divided into two equal portions with the upper segment containing all of the duodenum and jejunum. The number of adenomas arising in the upper (USI) and lower small intestine (LSI) was determined at time of sacrifice. Given that numbers are unchanged All procedures involving mice were carried out in accordance with the United Kingdom Animals (Scientific Procedures) Act 1986 and with guidance from local ethics committees on animal experimentation.

### Genotyping

Genotyping of *Apc^Min/+^* was performed as given earlier (24). Genome-wide microsatellite analysis was conducted using standard methods and involved the following markers: chromosome (chr) 1, *D1Mit430, 169, 132, 495, 17* and *292*; chr2, *D2Mit327, 395, 411, 229, 148,* and *230*; chr3, *D3Mit178, 51, 320* and *352*; chr 4, *D4Mit227, 18, 193, 348, 308* and *256*; chr5, *D5Mit387, 352, 201, 157, 188, 168* and *409*; chr6, *D6Mit1, 83, 123, 284, 287, 194, 15* and *198*; chr7, *D7Mit178, 117, 228, 276, 323, 101* and *362*; chr8, *D8Mit155, 124, 289, 292, 45, 211, 213* and *49*; chr9, *D9Mit250, 90, 285, 336, 355, 347, 201* and *151*; chr10, *D10Mit189, 106, 194, 31, 42, 95, 233* and *103*; chr11, *D11Mit216, 339, 177* and *179* ; chr 12, *D12Mit182, 91, 143* and *17*; chr13, *D13Mit115, 179, 142, 107, 260* and *78*; chr14, *D14Mit126, 127, 174, 60, 102* and *107*; chr15, *D15Mit100, 70* and *159*; chr16, *D16Mit182, 4, 5, 189* and *106*; chr17, *D17Mit143, 51, 180, 20* and *142*; chr18, *D18Mit222, 208, 49* and *4*; chr19, *D19Mit28, 106, 90, 103* and *6*. *Prkdc* was genotyped as given in (19). All available irradiated and un-irradiated mice were genotyped.

### Statistical analysis

The MapManager QTX program (25) was used to perform interval mapping (IM) and permutation testing (PT). Mapping of likeliest position of a QTL on a chromosome and the association with a given trait is determined by the statistic value described as the Likelihood Ratio Statistic (LRS) or the Log of Odds (LOD). The observed LRS was tested against the null hypothesis that the QTL did not occur by chance in the genome. Empirical significance thresholds (suggestive, significant and highly significant) were computed for each individual trait (USI and LSI) and empirical p values calculated for each observed LRS. IM analysis was also performed using Cartographer QTL (9). Composite interval mapping (CIM; (11,13,14) to investigate multiple significant peaks observed from IM was conducted in Cartographer.

### Selection and interrogation of target genes

Following the identification of the physical positions of each QTL, a comprehensive list of genes mapping to a region ±15 cM from the most likely position of the QTLs was collated using the Ensemble databases. These lists were checked for known tumour susceptibility loci, and searched for coding non-synonymous single nucleotide polymorphisms (CnSNP) between BALB and C57BL/6J using MPD database (26). All selected polymorphisms were verified by re-sequencing DNA taken from the mice used in the backcross. Sequencing was carried out on a 3100 ABI prism sequence analyser; primers and protocols are available on request.

### Bioinformatic tools used to predict functional influence of non-synonymous polymorphisms

The functional influences of CnSNPs were studied using bioinformatics software, including Polyphen (27) and SIFT (28). Sequence alignment and conservation of the SNPs were analysed by EMBL-EBI and Polyphen. A Search Tool for the Retrieval of Interacting Genes or Proteins (STRING) was used to identify whether the CnSNPs belonged to known or predicted functional domains (29). These domains were also confirmed using scientific literature searches.
